# Immunosuppressive Effect of B7-H4 Pathway in a Murine Systemic Lupus Erythematosus Model

**DOI:** 10.3389/fimmu.2017.01765

**Published:** 2017-12-11

**Authors:** Ze Xiu Xiao, Xu Zheng, Li Hu, Julie Wang, Nancy Olsen, Song Guo Zheng

**Affiliations:** ^1^Department of Clinical Immunology, Third Hospital at Sun Yat-sen University, Guangzhou, Guangdong, China; ^2^Laboratory of Immunotherapy, Sun Yat-Sen University, Guangzhou, China; ^3^Division of Rheumatology, Milton S. Hershey Medical Center at Penn State University, Hershey, PA, United States

**Keywords:** B7-H4, dendritic cell, autoimmunity, systemic lupus erythematosus, CD4^+^ T-cell

## Abstract

B7-H4, one of the co-stimulatory molecules of the B7 family, has been shown to play an important role in negatively regulating the adaptive immune response by inhibiting the proliferation, activation, and cytokine production of T cells. In this study, we investigate the role of B7-H4 in development of systemic lupus erythematosus (SLE). We investigated a murine model of SLE using transfer of bone marrow-derived dendritic cells (BMDCs) that were incubated with activated syngeneic lymphocyte-derived DNA. The recipient mouse produced anti-ds-DNA antibodies as well as displayed splenomegaly and lymphadenopathy as shown by significantly increased weights, and the kidneys showed lupus-like pathological changes include urine protein and glomerulonephritis with hyperplasia in glomeruli and increased mesangial cells and vasculitis with perivascular cell infiltration, glomerular deposition of IgG and complement C3. We showed that B7-H4 deficiency in BMDCs could cause greater production of anti-ds-DNA antibodies in transferred mice, and the lymph tissue swelling and the kidney lesions were also exacerbated with B7-H4 deficiency. Treatment with a B7-H4 antagonist antibody also aggravated the lupus model. Conversely, B7-H4 Ig alleviated the lupus manifestations. Therefore, we conclude that B7-H4 is a negative check point for the development of SLE in this murine model. These results suggest that this approach may have a clinical potential in treating human SLE.

## Introduction

Systemic lupus erythematosus (SLE) is a chronic inflammatory autoimmune disease that mainly affects women in their childbearing years (1). It is characterized by increased production of autoantibodies against nuclear and/or cytoplasmic antigens, immune complex deposition in the microvasculature of various organs, complement activation and integration, leukocyte infiltration in multiple organs, and various types of tissue damage (2–4). Immune complex accumulation in the kidney triggers a series of events that result in kidney inflammation and injury, and lupus nephritis is one of the most severe disease complications. Approximately 50–80% of lupus patients suffer from lupus nephritis and it is associated with SLE mortality (5–7). The etiology of SLE is not well understood, but it links to genetic susceptibility, hormonal modulation, and environmental factors and the guts microbe associated with diets (8), as well as to the most important immune disorders (9). Both B and T lymphocytes participate in lupus pathogenesis (10–12). Available treatments for SLE are generally effective in many patients, but it remains a disease without a cure and the treatments have significant side effects (13–17). Biological and immunotherapies for SLE, such as those targeting B cells (18), regulatory T cells (19, 20), and cytokines are available and others are in development.

The strain of C57BL/6 (B6) mice bearing the homozygous Fas-lpr/lpr mutation (B6-lpr/lpr) is an extensively studied murine model for SLE, in which mutation of the Fas apoptotic gene leads to a spontaneous autoimmune disorder that displayed similar with human SLE syndrome (21–23). Studies in this mouse strain recapitulate many aspects of human SLE such as anti-chromatin, anti-DNA, and anti-IgG serum autoantibodies as well as a polyclonal increase in total immunoglobulins (24). Disease severity in LPR mice is highly dependent on genetic background. For example, MRL-lpr/lpr mice produce high levels of IgG autoantibodies to DNA and develop a severe glomerulonephritis due to deposition of immune complexes, while B6-lpr/lpr mice produce low levels of autoantibodies with much milder immunopathology than MRL-lpr/lpr mice (21, 25). While this lupus murine strain displayed genetic mutation thus have certain differences with human SLE, Xiong and his colleagues have developed a model in which subcutaneous immunized syngeneic activated lymphocyte-derived DNA (ALD-DNA) to the syngeneic BALB/c mice could function as an autoantigen to induce a SLE syndrome, ALD-DNA is a kind of pathologic DNA produced from Con-A-induced apoptotic lymphocytes. It could be recognized by the antigen-presenting cells (APCs) and then activates the immune system, while the UALD-DNA (unactivated lymphocyte-derived DNA) gained from the normal lymphocytes is unable to activate immune system and induce the SLE syndrome (26–28). This lupus model can be induced in any strain of mice without genetic manipulation. However, they have subcutaneously immunized mice with ALD-DNA combined with adjuvant, raising a concern that the cell type responsible for ALD-DNA effects is not defined.

It has been well known that co-stimulatory pathways affect the development and progression of autoimmunity. The B7 family, one of most the important co-stimulatory molecular families, consists of many members that display different functions in the immune system (29). Previous studies have found that the B7-H1/PD-1 molecular pathway plays a critical role in negatively regulating Mog-induced EAE (30) and CTLA-4Ig controlled murine lupus nephritis (31). B7-H4 (B7S1) is another member of the B7 family, which was discovered in a human expressed sequence tagged database (32). The mRNA encoding B7-H4 is widely expressed in human and murine peripheral tissues, including kidney, liver, lung, spleen, thymus, and placenta (32, 33). By contrast, the expression of B7-H4 cell surface protein is limited, and it is not detectable in most human and murine tissues, except in normal human epithelial cells of the female genital tract, kidney, lung, and pancreas (34). Moreover, B7-H4 is detected widely in tumor tissues like renal cell carcinoma, lung cancer, ovarian cancer, melanoma, breast cancer, gastric cancer, pancreatic cancer, and liver cancer (35–42). B7-H4 expression can be induced by IL-6 and IL-10 on monocytes, macrophages, and myeloid dendritic cells (DCs), while GM-CSF and IL-4 can decrease B7-H4 expression by these cells (38, 43, 44). The receptor for B7-H4 is yet unknown. B- and T-lymphocyte attenuator was initially thought to be the receptor for B7-H4 (36), but further studies showed that this is not correct (45–47). The role of B7-H4 in the inhibition of immune responses has been shown in various *in vitro* systems (32, 33, 48, 49), and the suppressive function of B7-H4 *in vivo* is also well-studied (38, 50–52). Blockade of B7-H4 by a specific monoclonal antibody was shown to promote T cell responses in the presence of antigen stimulation *in vivo* (53), supporting a role for endogenous B7-H4 in suppression of the T cell response. However, a subsequent study using B7-H4 knockout (B7-H4-KO) mice revealed that B7-H4 is not a general inhibitor for T cell responses. B7-H4-deficient mice in a BALB/c background mounted slightly enhanced T-helper type 1T cell responses and displayed mildly lowered parasite burdens upon *Leishmania* infection compared to wild-type mice, suggesting a B7-H4 has an inhibitory, albeit mild, effect on Th1 responses (54). Others have also reported that the soluble B7-H4 in serum of lupus nephritis patients was strongly associated with serum creatinine levels (55), suggesting a possible correction between SLE and B7-H4 signal pathway.

On a C57BL/6 genetic background, B7-H4-KO mice did not show any signs of autoimmunity or disruption of immune cell homeostasis. B7-H4-KO mice display normal numbers and ratios of T cells, B cells, NK cells, and NKT cells and macrophages in flow cytometry analysis. In addition to T cells, B7-H4 has been shown to suppress proliferation of neutrophil progenitors, which play a profound role in controlling infection by *Listeria monocytogenes* (56). Upregulation of B7-H4 on APCs by IL-10 secreted by Tregs has been proposed as a potential inhibitory mechanism of regulatory T cells (44). B7-H4 Ig also can alleviate the disease of CIA (53) and moderate MOG (35–55)/CFA-induced EAE in C57BL/6 mice (57). However, the role of B7-H4 in SLE has not been thoroughly investigated.

In this study, we raised a new method of inducing the mice SLE model, by transferring bone marrow-derived dendritic cells (BMDCs) that incubated with ALD-DNA to mice through tail vein, and using this model explored the potential role of B7-H4 in SLE disease progression by blocking or increasing B7-H4 expression. The results demonstrate that the deficiency of B7-H4 in DCs in murine SLE exacerbated the disease and further, that the exacerbation is dependent on CD4^+^ T cells. Conversely, increasing B7-H4 in the lupus mice model ameliorated the disease. These findings imply that targeting B7-H4 could be a novel strategy for the treatment of patients with SLE and other autoimmune diseases.

## Materials and Methods

### Mice and Cells

#### Mice

Female BALB/c mice, C57BL/6 mice, and C57BL/6-lpr/lpr mice were purchased from Model Animal Research Center of Nanjing University, B7-H4-KO mice were generated in the investigator’s laboratory and have been backcrossed to B6 background for 10 generations. B6-lpr/lprxB7-H4-KO (H4-lpr) mice were obtained by backcrossing between B6-lpr/lpr and B7-H4-KO mice. All mice were housed in the Center of Experimental Animals of Sun Yat-sen University. The use of animals was approved by the IACUC committee at Sun Yat-sen University.

#### Cells

Cells include the following: BMDCs; P388d1: murine macrophage; GK1.5 cells: hybridoma for CD4^+^ T cells depletion; 6H3 cells: hybridoma for B7-H4 antagonist.

### Splenocytes Preparation

Spleens of BALB/c mice or C57 mice were aseptically removed and were triturated with two frosted glass slides immersing in PBS (Gibco, USA) in a plastic dish. Cells that passed through the filter screen were washed twice with PBS. The erythrocytes were lysed with ACK buffer and the remaining splenocytes were diluted to a final concentration of 2 × 10^6^ cells/ml and cultured in RPMI 1640 containing 10% heat-inactivated fetal bovine serum (Gibco), 100 IU/ml penicillin and 1% sodium pyruvate, and 1% Hepes (1 M).

### ALD-DNA Extraction and Purification

The splenocytes were stimulated with Con-A (5 µg/ml) to an apoptotic status for 6 days (58). Genomic DNA from syngeneic apoptotic splenocytes was treated with S1 nuclease (Takara Bio, Shiga, Japan) and proteinase K (Sigma-Aldrich) and then purified using the DNeasy Blood and Tissue Kits (Qiagen, Valencia, CA, USA) according to the manufacturer’s instructions. The concentration of DNA was determined by the Nano-drop. The final A260/A280 for the DNA preparations was more than 1.8. This preparation was the activated syngeneic lymphocyte-derived DNA (ALD-DNA).

### Preparation of BMDCs

The bone marrow cells were separated and prepared as single cell suspensions, erythrocytes were lysed with ACK buffer, and the remaining bone marrow cells were cultured in RPMI 1640 containing 10% heat-inactivated fetal bovine serum (Gibco), 100 IU/ml penicillin, 1% sodium pyruvate, and 1% hepes, and with 50 ng/ml rmGM-CSF, 2.5 ng/ml rmIL-4 to induce the differentiation of DCs. After 6 days, the DCs were identified using FACS staining with specific fluorescence CD11c antibody.

### DC Transfer

Bone marrow-derived dendritic cells were collected from the bone marrow cells as above. Groups of mice were adoptively transferred with BMDCs with or without incubating with 5 µg/ml ALD-DNA in complete medium for 12 h prior to injection. The BMDCs group was transferred with BMDCs alone that were free of ALD-DNA while BMDCs-ALD-DNA group was transferred with BMDCs that had been incubated with ALD-DNA. The dose of 5 × 10^5^ cells per mouse was administered by tail vein injection.

### Detection of Anti-ds-DNA Autoantibody

Anti-ds-DNA antibodies were detected by an ELISA. Briefly, 96-well plates were coated with 200 µg/ml salmon DNA overnight at 37°C. After washing four times with PBS containing 0.05% Tween-20 (PBST), the plates were blocked with 10% fetal bovine serum (Gibco) in PBS for 1 h, then 50-fold or 100-fold diluted in 1% BSA. The samples were added and incubated for 2 h at room temperature and washed once with PBST, then primed with horseradish peroxidase (HRP)-conjugated goat anti-mouse antibody. After washing seven times with PBST, the color development was primed with TMB (Sigma-Aldrich-T 0440), the reaction was stopped by 0.5 M H_2_SO_4_ and absorbance at 450 nm was measured in a microplate reader.

### *In Vivo* CD4^+^ T Cells Depletion

*In vivo* depletion of CD4^+^ T lymphocytes was achieved by using the monoclonal rat anti-mouse CD4 antibody (clone GK1.5). Mice were given GK1.5 antibody 1 mg per mouse every week until fifth week when the experiments ended.

### Flow Cytometry Analysis

Fluorescence was detected by flow cytometry and analyzed with Flow-jo 7.6.1. The cells were re-suspended in ice-cold PBS containing 1% FBS and stained with anti-CD3, anti-CD4, anti-CD8, anti-CD19, anti-CD11c, anti-CD11b, anti-Ly6G^+^ 6C, NK1.1, anti-CD25 antibodies (e-Bioscience). Intracellular staining of IL-2, IL-4, IFN-γ, and Foxp-3 was performed after 4 hours stimulation with PMA and ionomycin following the standard protocol (BD Biosciences).

### Cytokine Assay

Cell culture supernatants were collected from cell culture well plates at the indicated time points, sera were collected from the different groups of mice. IFN-γ, TNF-α, IL-2 IL-4, IL-6, and IL-10 concentration was measured by CBA kit (BD Biosciences).

### Hydrodynamic Injection

Mice were injected with B7-H4 IgV plasmid or control Flag IgV plasmid on day 3 and day 7 in the ALD-DNA induced lupus model. Hydrodynamic injection was performed as described previously (45). Briefly, 20 µg of plasmid DNA in 2 ml of PBS was injected into the mouse tail vein. Mouse survival was 100% after injection.

### Histological Assessments of Nephritis

Mice were sacrificed at indicated weeks post cell injection and the kidneys were removed, fixed in formalin, embedded in paraffin, sectioned, and stained with hematoxylin and eosin. For immune-histochemical evaluation of renal disease, mice were sacrificed at specified weeks. Kidneys were either fixed in formalin or snap-frozen in Tissue Tek for cryostat sectioning. Formalin-fixed tissues were embedded in paraffin, sectioned, and stained by the anti-mouse IgG -HRP or anti-mouse C3 antibody. Frozen sections were fixed in 100% acetone and 1% paraformaldehyde, and stained with FITC-conjugated anti-mouse IgG antibody.

### Statistical Analysis

Statistical significance was assessed using Student’s *t*-test, and the data were shown as mean ± SD unless otherwise noted. Statistical analyses of data were performed using the Graph-Pad Prism (version 4.0) statistical program. *P* < 0.05 was considered as significant.

## Results

### BMDCs Incubated with ALD-DNA Can Induce an SLE Syndrome in Mice

**Figure 1:**
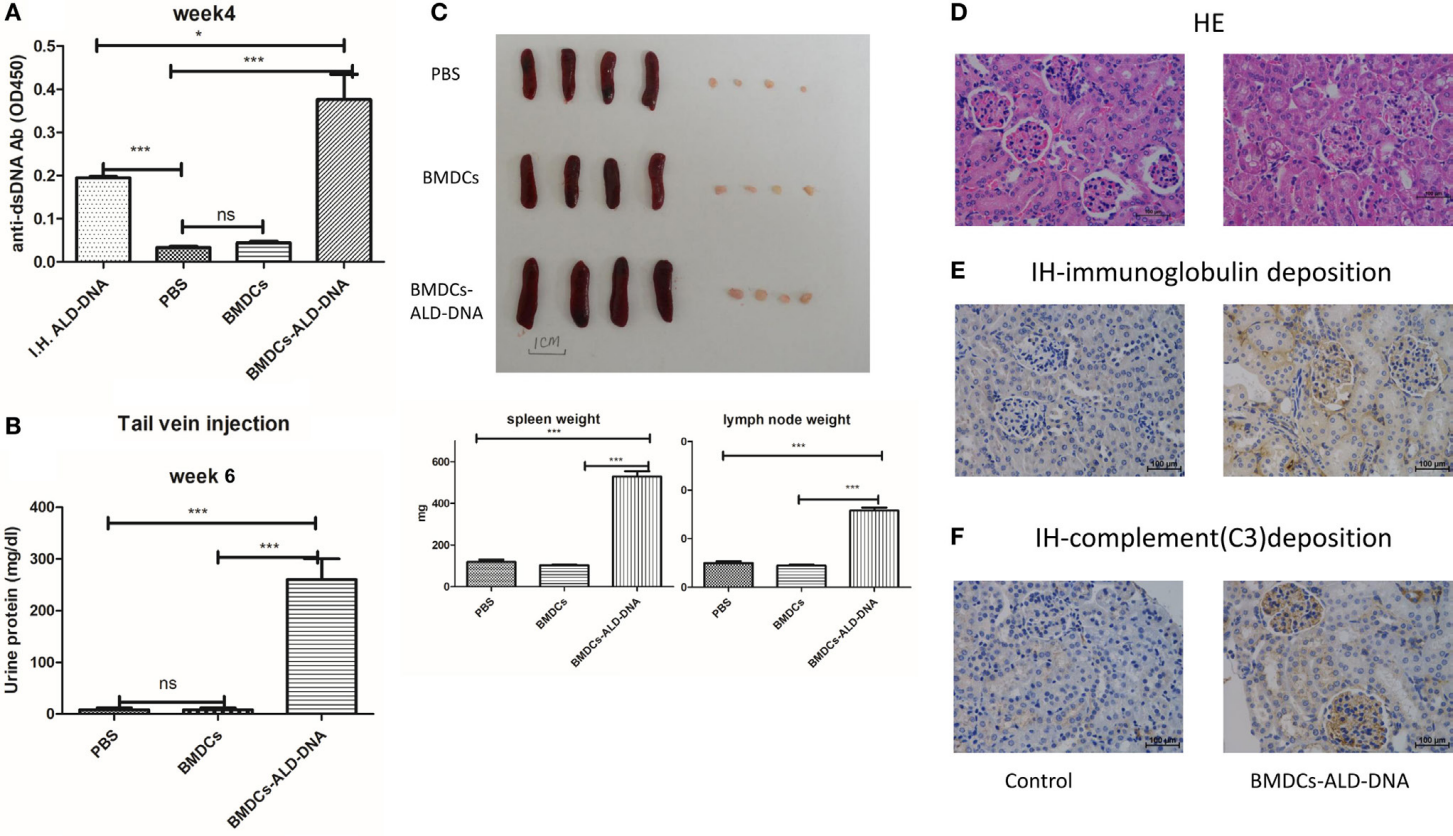
Bone marrow-derived dendritic cell (BMDC) incubated with activated lymphocyte-derived DNA (ALD-DNA) induced mice lupus symptom. **(A)** Total IgG against ds-DNA in sera from the mice that transferred with: (left to right) positive control group (I.H.ALD-DNA): subcutaneously immunized ALD-DNA with adjuvant; negative control group: PBS, BMDCs; experimental group: BMDCs-ALD-DNA. **(B)** Urine protein levels of lupus mice on week 6 post BMDCs-ALD-DNA or the control substances transferred were assessed by visual urine test paper. **(C)** The gross appearance and the weight of spleens or lymph nodes from the control group mice and the experimental group mice, the mice were sacrificed on week 20 post BMDCs-ALD-DNA or the control substances transferred to get the spleens and the kidneys. **(D–F)** Histology **(D)** and total IgG **(E)** or C3 **(F)** glomerular deposition of kidney from the control group and the experimental group mice. The mice were sacrificed on week 20 after the BMDCs or the control substances transferred. Representative result from each group of five mice is shown. Values represent means ± SEM (*p < 0.05, ***p < 0.0005). Scale bar: 100 µm.

Xiong and his colleagues had previously demonstrated that subcutaneous immunization of mice with activated syngeneic lymphocyte-derived DNA (ALD-DNA) could successfully induce murine SLE syndromes manifested by high levels of anti-ds-DNA antibody (26, 28, 59). We confirmed that after subcutaneous immunization with ALD-DNA (Figure S1A in Supplementary Material), the mice displayed high levels of anti-dsDNA antibodies in the 2 weeks after immunization, and the kidney showed glomerulonephritis at 20 weeks after immunization (Figure S1B in Supplementary Material). As we know that DCs are professional APC and the DCs expressing TLRs richly, for which is mainly recognized pathologic DNAs and presenting to immune system (60). There are many lines of evidence to indicate that DCs play a critical role in the development of SLE (61–71). To confirm the hypothesis that the ALD-DNA induced SLE syndrome was mediated by DCs, BMDCs incubated with ALD-DNA (BMDCs-ALD-DNA) were transferred to normal female C57BL/6(B6) mice. The transferred BMDCs-ALD-DNA successfully induced the SLE-like syndrome in these transferred mice (ALD-DNA lupus model). The serum levels of anti-ds-DNA Abs were higher in mice that received BMDCs-ALD-DNA than in mice receiving ALD-DNA subcutaneous immunization which is lupus model positive control, while the mice that received PBS or BMDCs alone were unable to generate autoantibodies (Figure 1A). On week 6 after the BMDCs-ALD-DNA transfer, the lupus mice appeared urine protein (Figure 1B). Interestingly, we found the ALD-DNA directly transferred to the mice systemic vascular circulation through tail vein could not induce anti-dsDNA antibody production (Figure S1C in Supplementary Material). This might be due to the fact that ALD-DNA is a big molecular substance that caused immunotolerance and soon be metabolized. The BMDCs-UALD-DNA, a DNA derived from BMDCS that had been incubated with unactivated lymphocytes, was unable to induce the anti-dsDNA antibody after transfer, being consistent with previous report that normal DNA was unable to induce mice SLE syndrome (Figure S1D in Supplementary Material) (26–28). Moreover, the macrophage cells (P388D1) incubated with ALD-DNA and transferred to isogenic mice failed to induce autoantibody production like BMDCs do (Figure S1E in Supplementary Material). We found that after the BMDCs were incubated with ALD-DNA, the BMDCs displayed analogous changes in the cells when incubated with LPS or TLR9 ligand CPG (Figure S1F in Supplementary Material). These data showed that DCs are able to combine ALD-DNA and they together induced autoantibodies in mice. The mice that received BMDCs-ALD-DNA developed splenomegaly and lymphadenopathy as shown by significantly increased weights compared to the control group on week 20 after the BMDCs-ALD-DNA transferred (Figure 1C), and the total numbers of splenocytes and total T or B cells and NK cells showed differences in the BMDCs-ALD-DNA transferred mice compared to control mice (Figure S1G in Supplementary Material). Moreover, the BMDCs-ALD-DNA induced mice displayed glomerulonephritis with hyperplasia in glomeruli and increased mesangial cells compared with control mice (Figure 1D), the kidney from the model mice also appeared vasculitis with perivascular cell infiltration, glomerular deposition of IgG (Figure 1E), and complement C3 (Figure 1F). By contrast, control group mice had no kidney pathology. In addition, the lupus-like syndrome is dose dependent with BMDCs-ALD-DNA transferred to mice demonstrated by sera anti-ds DNA Abs levels and the size changes of spleens and lymph nodes (Figures S1H,I in Supplementary Material). Above results demonstrate that ALD-DNA could induced the murine SLE syndrome through BMDCs, and the severity of lupus is positively correlated with the numbers of BMDCs-ALD-DNA transferred.

### B7-H4 Deficient in DCs Exacerbated the BMDCs-ALD-DNA Lupus Model Depends on CD4^+^ T Cells

**Figure 2:**
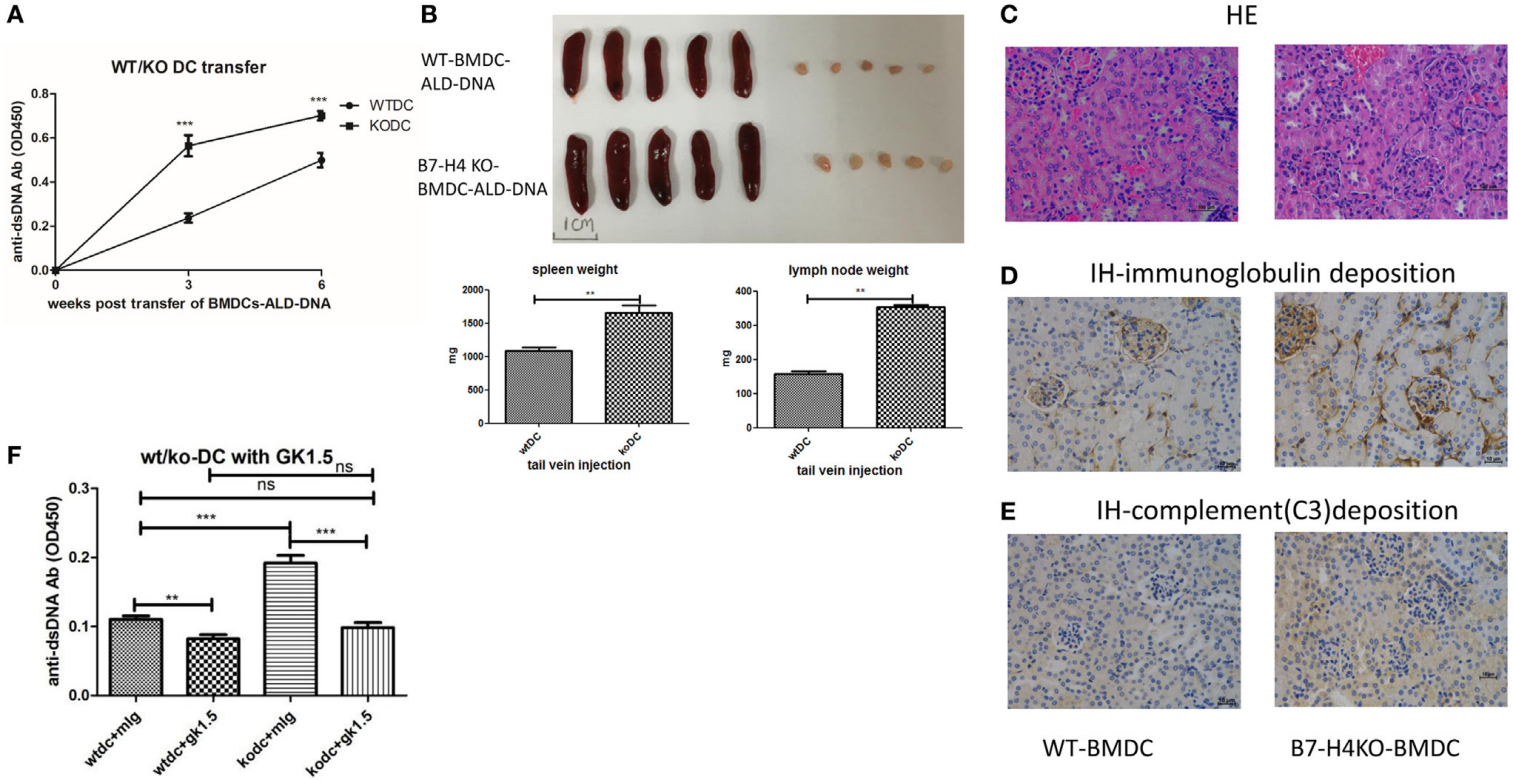
B7-H4-deficient bone marrow-derived dendritic cells (BMDCs) exacerbated activated lymphocyte-derived DNA (ALD-DNA) lupus model dependent on CD4^+^ T cells. **(A–E)** Total IgG against ds-DNA in sera **(A)** and the gross appearance and the weights **(B)** of spleens or lymph nodes and histology **(C)** and total IgG **(D)** or C3 **(E)** glomerular deposition of kidney from the mice that transferred with wt BMDCs-ALD-DNA or B7-H4 knockout (B7-H4-KO) BMDCs-ALD-DNA. The mice were sacrificed on week 20 after the WT or the B7-H4-KO BMDCs-ALD-DNA transferred to get the spleens and the kidneys. **(F)** Total IgG against ds-DNA in sera on week 4 of the lupus mice that depleted the CD4^+^ T cells with GK1.5 in advance before transferred BMDCs, the GK1.5 antibody was given to lupus mice 2 weeks before the BMDCs transferred till to week 4 post the BMDCs transferred. Representative result from each group of five mice is shown. Values represent means ± SEM (**p < 0.005, ***p < 0.0005). Scale bar: 100 µm.

The BMDCs-ALD-DNA lupus model provides an ideal tool to study the effect of co-stimulatory molecules expressed on DCs and receptors on T cells in lupus. To test the hypothesis that B7-H4 plays a negative role in lupus pathogenesis and development, we transferred BMDCs isolated from B7-H4-KO or wild-type mice into normal recipient mice. We observed that the levels of anti-ds-DNA antibodies were higher in mice receiving B7-H4-KO BMDCs-ALD-DNA than in mice that received wide-type normal BMDCs-ALD-DNA (Figure 2A). Nonetheless, the B7-H4-KO BMDCs free of ALD-DNA transferred to mice failed to induce anti-dsDNA antibody (Figure S2A in Supplementary Material).

Accordingly, B7-H4-KO-BMDCs-ALD-DNA induced lupus mice displayed more splenomegaly and lymphadenopathy (Figure 2B), as well as kidney lesions with the deposition of immunoglobulin and complement C3 (Figures 2C–E). It has been suggested that CD4^+^ T cells play prominent roles in the pathogenesis of lupus (72–76). Xiong’s studies also verified that the production of anti-ds-DNA Abs in the ALD-DNA lupus model was dependent on CD4^+^ T cells (77). We examined the components of the inflammatory cells in the lupus mice and found that the percentages of CD4^+^ T, IL-2^+^, IL-17^+^ cells in peripheral blood were significantly higher in B7-H4-KO BMDCs-ALD-DNA induced lupus mice than that in WT BMDCs-ALD-DNA induced lupus mice (Figure S2B in Supplementary Material). Conversely, the anti-inflammation cells CD4^+^Foxp3^+^ Treg cells (Figure S2C in Supplementary Material) were significantly lower in B7-H4-KO BMDCs-induced lupus mice compared to control mice; thus, these results show that the lack of B7-H4 on DCs has changed the balance between T effector and Treg cells in this model. Given that pro-inflammatory cytokines are crucial in lupus pathogenesis (78, 79), we also measured the cytokine production level *in vitro* that WT or B7-H4-KO BMDCs incubated with ALD-DNA and then co-cultured with wild-type normal CD4^+^ cells supernatants used a cytokine test kit named CBA (BD). We observed that co-cultured cells displayed higher levels of secreted cytokines (IL-2, IL-17, IL-6, TNF-α, and IFN-γ) in B7-H4-KO BMDCs compared with WT BMDCs (Figure S2D in Supplementary Material), although the co-cultured cell proliferation showed no differences between two groups (Figure S2E in Supplementary Material). The cytokines in the sera from the WT BMDCs-ALD-DNA or B7-H4-KO BMDCs-ALD-DNA induced lupus mice also showed the comparable differences (Figure S2F in Supplementary Material). Since exacerbation of the lupus syndrome in the B7-H4-deficient BMDCs-ALD-DNA induced model can be abolished by CD4^+^ T cell depletion *in vivo* (Figure 2F), thus this suggests that the B7-H4 signal mainly affects CD4^+^ T cell function to affect lupus disease manifestations. These data also implicate endogenous B7-H4 as a checkpoint molecule in suppressing ALD-DNA-induced lupus model.

### B7-H4 Antagonist Antibody Treatment Increased Disease Manifestations in the BMDCs-ALD-DNA Lupus Model

**Figure 3:**
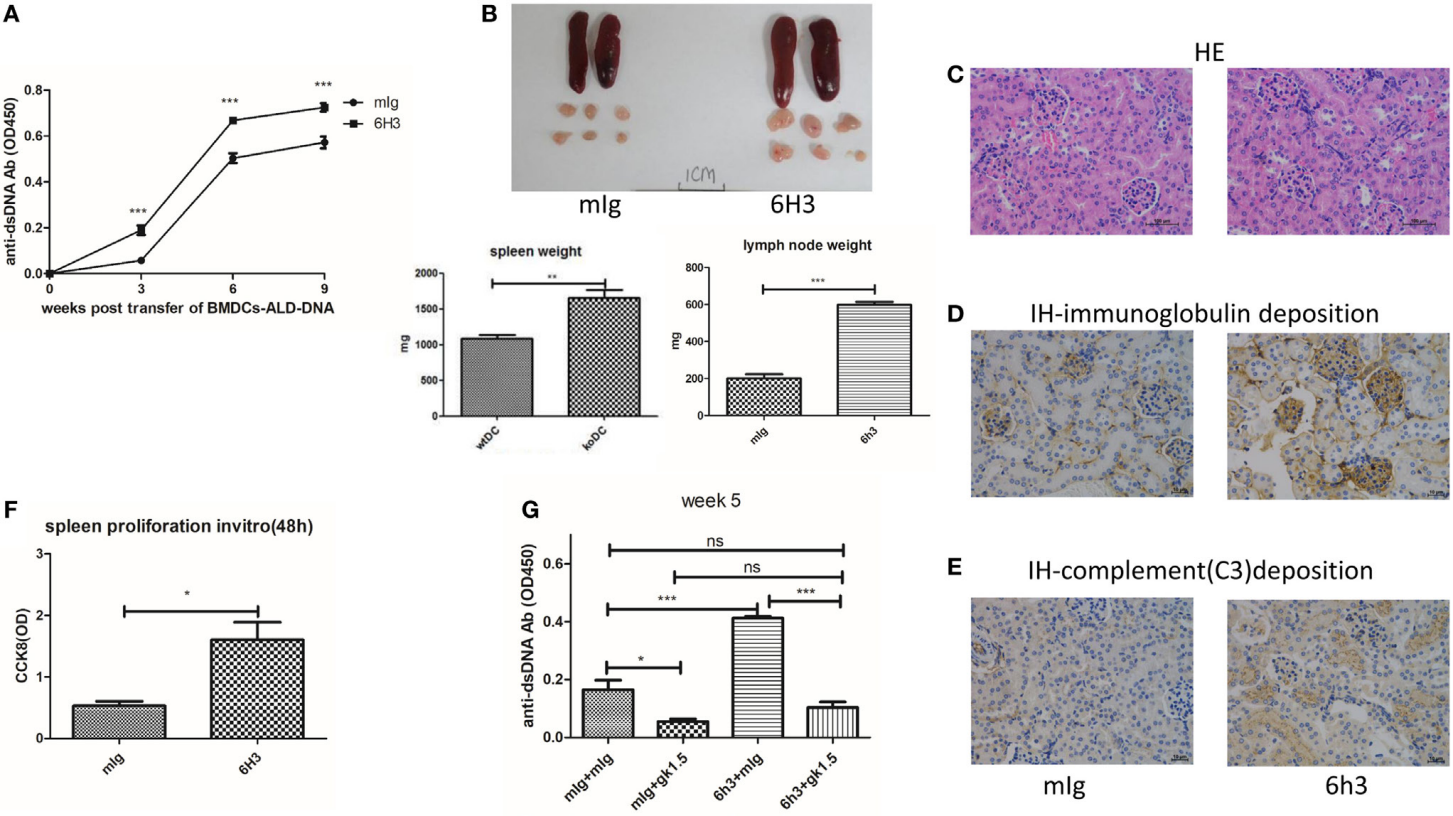
B7-H4 antagonist antibodies aggravated bone marrow-derived dendritic cell (BMDC)-activated lymphocyte-derived DNA (ALD-DNA) induced lupus model. **(A–E)** Total IgG against ds-DNA in sera **(A)** and the gross appearance and the weights **(B)** of spleens or lymph nodes and histology **(C)** and total IgG **(D)** or C3 **(E)** glomerular deposition of kidney from the mice that transferred with wt BMDCs-ALD-DNA, the lupus mice were intraperitoneal injected with mouse Ig or 6H3 on the same day after the BMDCs transferred. The mice were sacrificed to get the spleens, lymph nodes, and kidneys on week 20 after the BMDCs transferred. **(F)** 6H3 promoted the proliferation of splenocytes from lupus mice with the stimulation of ALD-DNA *in vitro*. **(G)** Total IgG against ds-DNA in sera on week 5 of the lupus mice that depleted the CD4^+^ T cells with GK1.5 in advance before transferred BMDCs, the GK1.5 antibody was given to lupus mice 2 weeks before the BMDCs transferred till to week 5 post the BMDCs transferred. Representative result from each group of five mice is shown. Values represent means ± SEM (*p < 0.05, **p < 0.005, ***p < 0.0005). Scale bar: 100 µm.

To further validate the role of B7-H4 in lupus pathogenesis, we then tested effects of B7-H4 antibody named 6H3 on lupus development and progression in this induced lupus model. 6H3 bound to B7-H4 transfected on 293T cells (Figure S3A in Supplementary Material). We revealed that 6H3 infusion significantly increased the levels of the anti-ds-DNA antibodies in mice receiving BMDCs-ALD-DNA (Figure 3A). The spleen and lymph nodes from the lupus mice with 6H3 treatment were also larger than those of the groups treated with control mIg (Figure 3B). The total numbers of splenocytes show parallel differences, as well as the T, B, and DC subsets in the spleens were shown differences between the 6H3 treatment and control group (Figure S3B in Supplementary Material). The CD4^+^ T cells and IL-2^+^CD4^+^ T cells ratio were significantly higher in the 6H3 group while the CD4^+^Foxp3^+^ T cells decreased in the 6H3 treatment compared to control group (Figure S3C in Supplementary Material). The kidney lesion (Figure 3C) as well as the deposition of immunoglobulin (Figure 3D; Figure S3D in Supplementary Material) and complement C3 (Figure 3E) were more evident in 6H3 treatment group than in mIg treatment group. The proliferation of lupus mice splenocytes was significantly higher in 6H3 treatment than in control mIg treatment mice *in vitro* (Figure 3F). The effect of 6H3 in the BMDCs-ALD-DNA induced lupus model was diminished by CD4^+^ T cell depletion (Figure 3G), in accordance with B7-H4-KO-BMDCs-ALD-DNA-induced sever lupus mice syndrome dependent on CD4^+^ T cells (Figure 2F). These data provide further evidence that B7-H4 deficiency could reinforce the BMDCs-ALD-DNA lupus model and the exacerbation dependent on CD4^+^ T cells.

### Suppression of BMDCs-ALD-DNA Induced Lupus Model by B7-H4Ig

**Figure 4:**
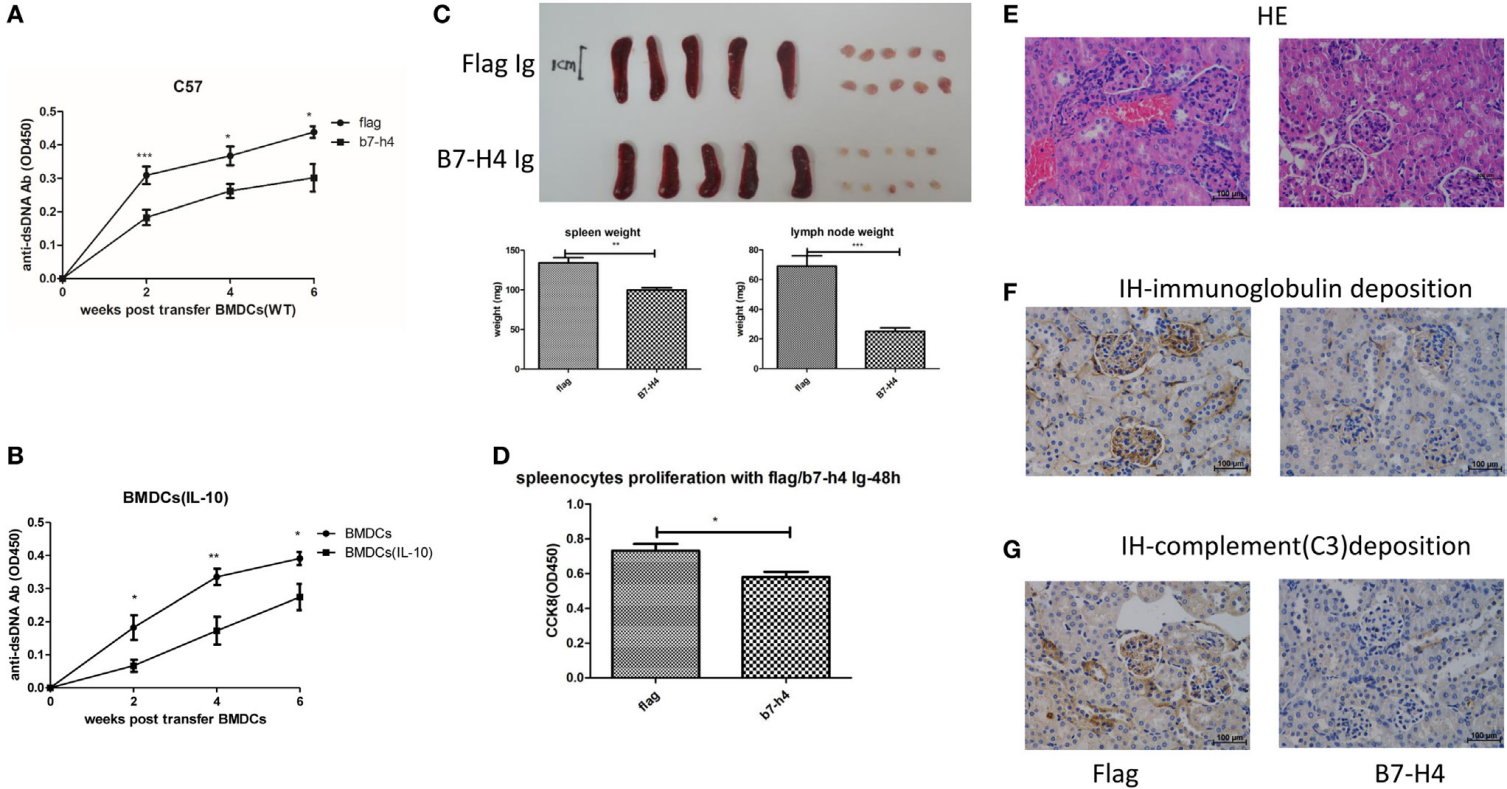
Suppression of bone marrow-derived dendritic cells (BMDCs)-activated lymphocyte-derived DNA (ALD-DNA) induced lupus model by B7-H4Ig. **(A)** Total IgG against ds-DNA in sera from the mice that transferred with wt BMDCs-ALD-DNA, the lupus mice were treated with flag or B7-H4Ig twice after the dendritic cells (DCs) transferred. **(B)** Total IgG against ds-DNA in sera from the mice that transferred with wt BMDCs-ALD-DNA, the BMDCs were pre-presented on rmIL-10 or not. **(C)** The gross appearance and the weights of spleens or lymph nodes from the mice that transferred with wt BMDCs-ALD-DNA, the lupus mice were treated with flag or B7-H4Ig twice after the DCs transferred, the mice were sacrificed on week 20 after the cells transferred to get the spleens and the kidneys. **(D)** B7-H4Ig inhibited the proliferation of splenocytes from lupus mice with the stimulation of ALD-DNA *in vitro*. **(E–G)** The histology **(E)** and total IgG **(F)** or C3 **(G)** glomerular deposition of kidney from the mice that transferred with wt BMDCs-ALD-DNA, the lupus mice were treated with flag or B7-H4Ig twice after the DCs transferred. Representative result from each group of five mice is shown. Values represent means ± SEM (*p < 0.05, **p < 0.005, ***p < 0.0005). Scale bar: 100 µm.

While our data show that B7-H4 deficiency or B7-H4 antagonist antibody 6H3 could aggravate the BMDCs-ALD-DNA lupus model, a potential approach to inhibit the progression of lupus is by regulating B7-H4 expression in the form of an agonist of the unknown receptor (53). Liu and his colleagues reported that hydrodynamic tail vein injection of targeted DNA plasmid in the mouse resulted in specific plasmid protein overexpression (80). We adopted this method and hydrodynamically injected B7-H4IgV plasmid *via* tail vein to the mouse (C57BL/6 or BALB/c background) and tested the B7-H4 confusion protein expression in serum. As shown in Figure S4A in Supplementary Material, the B7-H4Ig was expressed in a concentration peak 24 h post injection and disappeared at around 120 h post injection. The BMDCs-ALD-DNA induced lupus model mice were injected with B7-H4IgV plasmid or control Flag plasmid at 2 days and 5 days after BMDCs-ALD-DNA transfers. Unlike the control Flag group, B7-H4Ig treatment markedly suppressed the production of total IgG of anti-ds-DNA antibodies, and the suppression effect appeared on normal wild-type C57BL/6 mice (Figure 4A) or BALB/c mice (Figure S4B in Supplementary Material) or B7-H4-KO mice (Figure S4C in Supplementary Material). Moreover, we revealed that B7-H4 Ig treatment also neutralized the differences in the autoantibody levels in the B7-H4KO BMDCs-ALD-DNA and BMDCs-ALD-DNA induced autoantibodies (Figure S4D in Supplementary Material). Kryczek and his colleagues have previously found that B7-H4 expression could be increased on DCs by rmIL-10 (44). As shown in Figure S4E in Supplementary Material, B7-H4 mRNA expression level was relatively increased in BMDCs as rmIL-10 was present. The BMDCs-ALD-DNA that treated with rmIL-10 induced lower anti-ds-DNA antibody level compared with the BMDCs-ALD-DNA group without rmIL-10 treatment (Figure 4B). Meanwhile, the B7-H4Ig treated lupus mice displayed relatively mild splenomegaly and lymphadenopathy compared to mice treated with control Flag Ig (Figure 4C). The pro-inflammatory cell infiltrate tended to be lower in B7-H4Ig mice compared with control Flag Ig mice, while the regulatory CD4^+^Foxp3^+^ T cells were increased in the mice treated with B7-H4Ig (Figure S4F in Supplementary Material). *In vitro*, splenocytes proliferation from ALD-DNA stimulation of the BMDCs-ALD-DNA induced lupus model was lower in B7-H4Ig present compared with the control Flag present (Figure 4D). The renal lesion in these mice was milder with B7-H4Ig treatment compared with control Flag group (Figures 4E–G). Thus, our data have demonstrated that B7-H4Ig treatment alleviates the severity of BMDCs-ALD-DNA induced lupus-like syndrome, and it might potentially be clinically valuable in treating SLE.

### B7-H4-Deficient C57BL/6-lpr/lpr Mice Developed Exacerbation of Lupus

**Figure 5:**
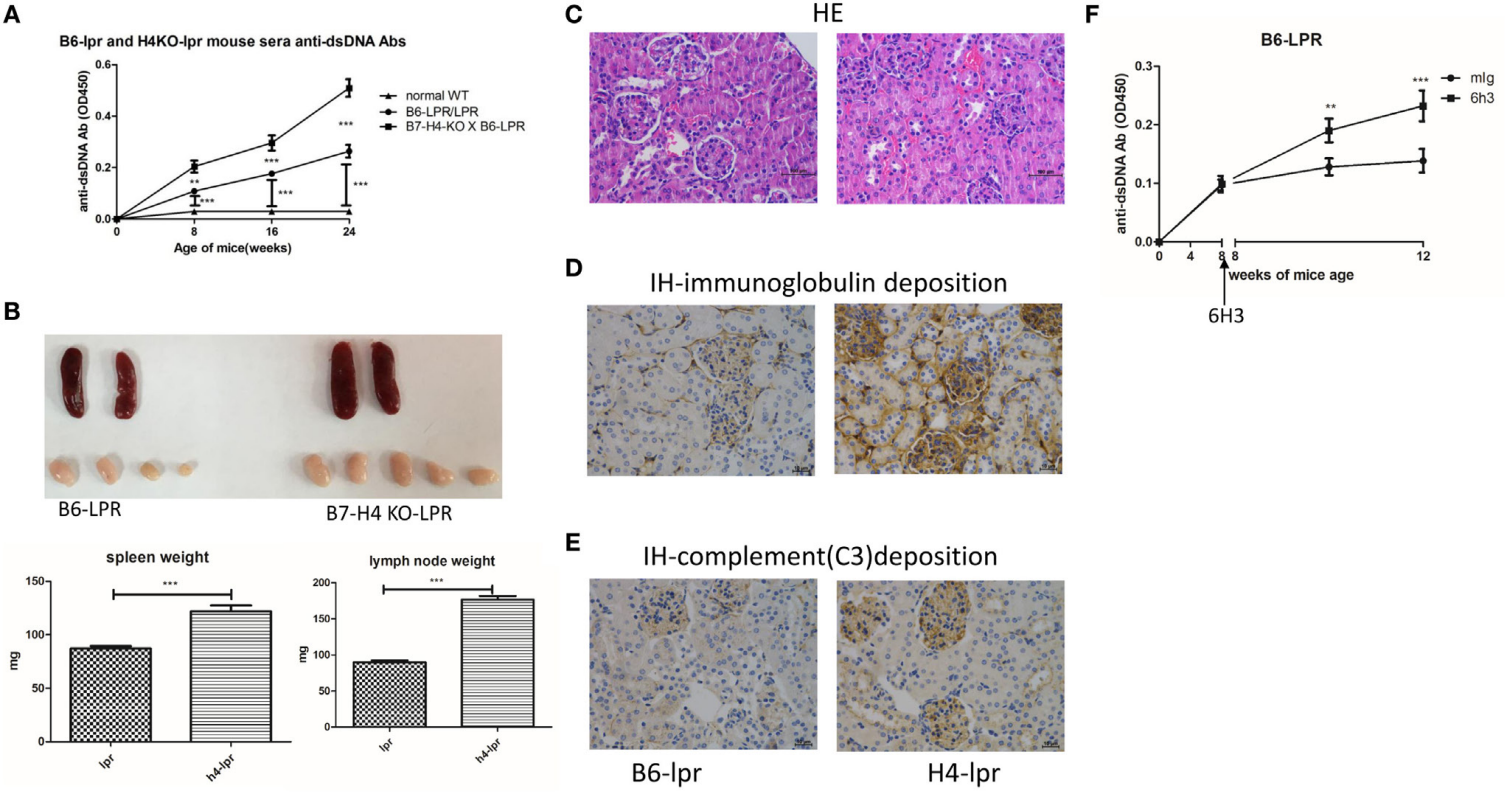
B7-H4-deficient C57BL/6-lpr/lpr mice develop exacerbated lupus symptom. **(A–E)** Total IgG against ds-DNA in sera **(A)** and the gross appearance and the weight **(B)** of spleens or lymph nodes and histology **(C)** and total IgG **(D)** or C3 **(E)** glomerular deposition of kidney from the C57BL/6-lpr/lpr mice and the B7-H4 knockout (B7-H4-KO) lpr mice. **(F)** Total IgG against ds-DNA in sera of the C57BL/6-lpr/lpr mice with mouse Ig or 6H3 treatment. The mice were sacrificed on week 30 post BMDCs-ALD-DNA or the control substances transferred to get the spleens and the kidneys. Representative result from each group of five mice is shown. Values represent means ± SEM (**p < 0.005, ***p < 0.0005). Scale bar: 100 µm.

We also used the B6-lpr/lpr strain that is considered as a commonly used mouse model for SLE to validate the effect of B7-H4 on lupus disease. The B6-lpr mice were back crossed with B7-H4-KO mice to produce B7-H4-KO lpr mice. Consistently, increased anti-ds-DNA Ab production was observed in H4-lpr mice (B7-H4-KO lpr mice) compared to B6-lpr mice (Figure 5A). Moreover, the autoantibodies appeared significantly earlier in H4-lpr mice than in control mice. The H4-lpr mice developed greater splenomegaly and lymphadenopathy with significantly increased organ weights compared to B6-lpr mice (Figure 5B). Accordingly, the total number of splenocytes from H4-lpr mice was much more than from B6-lpr (Figure S5A in Supplementary Material). Increased inflammation cell subsets in H4-LPR mice were also observed compared to B6-lpr mice, the percentages of CD3^+^CD4^+^ T cells, CD3^+^CD4^−^CD8^−^ T cells, CD19^+^ B cells, NK1.1^+^ NK cells, and CD3^+^NK1.1^+^ NKT cells in peripheral blood were significantly higher in H4-lpr mice than in B6-lpr mice (Figure S5B in Supplementary Material), similarly, the follicular helper cell (Tfh) showed significantly greater in the H4-lpr mice spleen compared to control (Figure S5C in Supplementary Material). Conversely, the CD4^+^Foxp3^+^ Treg cells in the peripheral blood were significantly lower in H4-lpr mice than in B6-lpr mice (Figure S5C in Supplementary Material). The H4-lpr mice also developed more severe glomerulonephritis with hyperplasia in glomeruli and increased mesangial cells than B6-lpr mice at ages of 30 weeks (Figure 5C). Furthermore, the H4-lpr mice displayed vasculitis with perivascular cell infiltration, glomerular deposition of IgG (Figure 5D) and complement C3 (Figure 5E), by contrast, B6-lpr mice had minor kidney lesion pathology. In addition, the B7-H4 antibody, 6H3 treatment increased the anti-ds-DNA antibodies B6-lpr mice (Figure 5F), while this antibody did not change the anti-ds-DNA Ab levels in normal C57BL/6 mice (Figure S5D in Supplementary Material). These data provide evidence that the B7-H4 signal also affects disease development in B6-LPR mice, it is furthermore indicated the role of B7-H4 in SLE.

## Discussion

Previous studies have demonstrated that activated syngeneic lymphocyte-derived DNA (ALD-DNA), a kind of pathologic nucleic acid that is hypomethylation separate from apoptotic lymphocytes, subcutaneous immunization can cause a lupus-like syndrome including autoantibodies and characteristic kidney lesions (26, 59, 81). Given that the professional APC, DCs play critical roles in the development of lupus (61–66), we hypothesized that ALD-DNA induced SLE syndrome was primarily mediated by DCs. To test this hypothesis, we transferred BMDCs incubated with ALD-DNA (BMDCs-ALD-DNA) to C57BL/6 (B6) mice and found that high titers of anti-ds-DNA antibodies developed, and the mice displayed the splenomegaly and kidney lesion that is similarly to lupus syndromes. Interestingly, the macrophage cannot induce lupus-like syndrome in the same manner, it is likely that TLR9 signal plays an important role in the ALD-DNA induced the activation of immune system and lupus model (59), since previous study has revealed that TLR9 is not expressed on macrophage (60). Thus, we have improved the induced lupus model that could be useful in studying the pathogenesis of lupus.

The data presented in this paper are also the first demonstration of the role of B7-H4 in regulating the development of murine ALD-DNA induced SLE. Using different strategies including B7-H4-deficient DCs, B7-H4 antagonist antibody 6H3 or B7-H4 confusion protein, we have demonstrated that B7-H4 expressed on DC could bind to its unidentified receptor on T cells to deliver a negative signal to suppress immune responses. As B7-H4-deficient mice do not show autoimmunity (56), B7-H4 signal deficiency markedly accelerates the development of lupus in a DC-induced lupus model, suggesting that the B7-H4 signal is crucial in the pathogenic condition and alleviates the BMDCs-ALD-DNA induced lupus progression. Although the receptor for B7-H4 is not yet identified, CD4^+^ T cells are crucial since the effect of B7-H4 signal on lupus development is diminished by CD4^+^ cell deletion. We developed the idea that B7-H4 may work through delivering a sustained negative signal to its putative receptor on CD4^+^ T cells. We also used the B6-lpr mice to further validate that the effect on B7-H4 on ALD-DNA induced lupus can be repeated in another commonly used model. Similarly, the blockade of the B7-H4 signaling pathway exacerbated lupus diseases, further demonstrating that this pathway is a negative signal to control immune responses in lupus.

Although the underlying mechanisms are not completely clear, the effect of B7-H4 on T cell subset function is likely. Chen and his colleagues previously reported that B7-H4 inhibits cell cycle progression from G0 to G1 in T cells upon T cell receptor engagement (32). Thus, the delay of T cell activation and proliferation could contribute to suppressive effects on lupus symptoms. We have observed that B7-H4-deficient B6-lpr mice had significantly increased inflammation cellularity in spleen and peripheral blood, and the predominant cell types are CD3^+^CD4^−^CD8^−^ T cells that produce Th1 and Th17 like cytokines. These so-called double negative T cells have been shown to contribute to the pathogenesis and progression of the B6-lpr phenotype (80).

Treg cells have been shown to suppress murine lupus-like syndromes and other autoimmune diseases (19, 20, 82), and the relationship between co-stimulatory signals and Treg subset development has been reported previously (83). Our results showed that the CD4^+^Foxp3^+^ Treg cells were decreased in B7-H4-KO DCs incubated with ALD-DNA-induced lupus mice and BMDCs-ALD-DNA mice treated with 6H3, a B7-H4 antibody. Additionally, Treg cells were also decreased in H4-lpr mice compared with the B6-lpr mice. It is likely that B7-H4 expressed on DCs binds to unidentified receptor and then promotes the Treg subset induction and function. The lack of B7-H4 could decrease Treg cells and then contribute to the exacerbation of the lupus disease. Others have reported that the B7-H4 Ig improved the EAE syndrome *via* IL-10/Tregs mechanisms (57). It warrants a further study whether the IL-10 also plays a role in B7-H4 signal in lupus. We also observed that the B7-H4 pathway affects proliferation of the ALD-DNA induced T cells but not normal T cells (Figure S6 in Supplementary Material), so it is likely that the B7-H4 pathway mainly affects the proliferation of T cells that have altered thresholds, particularly in an autoimmune disease condition.

Taken together, our results suggest that B7-H4 expressed on DCs plays an important role in negative regulation of autoimmunity in lupus mice through bonding the putative receptor on CD4^+^ T cells and influencing the regulatory T cells (Figure S7 in Supplementary Material). Targeting B7-H4 may provide a potentially and clinically valuable approach to treating patients with SLE and other autoimmune diseases.

## Author Contributions

XZX and ZX contributed equally as co-first author. SGZ designed the research; ZX, XZ and LH performed the experiments; ZX and JW analyzed the data; ZX, XZ, ON and SGZ wrote the manuscript. All the authors read and approved the final manuscript.

## Conflict of Interest Statement

The authors declare that the research was conducted in the absence of any commercial or financial relationships that could be construed as a potential conflict of interest.

## Supplementary Material

Figure S1 **(A)** Flow chart for the normal C57BL/6 mice immunized subcutaneously with activated lymphocyte-derived DNA (ALD-DNA). **(B)** Total IgG against ds-DNA in sera (left) and the histology of the kidney (right) from the mice that immunized subcutaneously with PBS or ALD-DNA. **(C)** Total IgG against ds-DNA in sera of normal wild-type C57BL/6 mice transferred with PBS, bone marrow-derived dendritic cells (BMDCs), ALD-DNA, or BMDCs-ALD-DNA through tail vein on week 4 post transferred. **(D)** Total IgG against ds-DNA in sera of normal wild-type C57BL/6 mice transferred with PBS, BMDCs-UALD-DNA, or BMDCs-ALD-DNA on week 4 post transferred. **(E)** Total IgG against ds-DNA in sera of normal wild-type C57BL/6 mice transferred with PBS, BMDCs-ALD-DNA, or P388D1-ALD-DNA. **(F)** The changes of the BMDCs after incubated with ALD-DNA, LPS or the widely accepted TLR9 ligand CPG for 24 h using FACS. **(G)** Total splenocytes and cell subsets numbers in the spleen between control mice and the BMDCs-ALD-DNA transferred mice. **(H)** Total IgG against ds-DNA in sera and the spleen or lymph node weight **(I)** of normal WT C57BL/6 mice transferred with BMDCs-ALD-DNA in different quantities (0, 1.25 × 105, 5 × 105, 10 × 105). Representative result from each group of five mice is shown. Values represent means ± SEM (**p* < 0.05, ***p* < 0.005, ****p* < 0.0005).

Figure S2 **(A)** Total IgG against ds-DNA in sera of normal wild-type C57BL/6 mice transferred with PBS, bone marrow-derived dendritic cells (BMDCs), B7-H4 knockout (B7-H4-KO) BMDCs, BMDCs-activated lymphocyte-derived DNA (ALD-DNA), or B7-H4-KO BMDCs-ALD-DNA on week 4 post transferred. **(B)** The component of inflammation cell subsets ratio and the FACS of the Tregs **(C)** in lupus mice transferred with wt BMDCs-ALD-DNA or B7-H4-KO BMDCs-ALD-DNA. **(D)** The cytokines secretion and the proliferation **(E)** of the wt BMDCs-ALD-DNA or B7-H4-KO BMDCs-ALD-DNA co-cultured with wild-type normal CD4^+^ T cells. **(F)** The cytokines secretion of the serum from the wt BMDCs-ALD-DNA or B7-H4-KO BMDCs-ALD-DNA-induced lupus mice. Representative result from each group of five mice is shown. Values represent means ± SEM (**p* < 0.05, ***p* < 0.005, ****p* < 0.0005).

Figure S3 **(A)** 6H3 bond B7-H4 that transfected on 293T cell. **(B)** The total splenocytes and other inflammation cells number of spleen and **(C)** the inflammation cells or the Treg cells ratio in the peripheral blood. **(D)** The immunofluorescent staining total IgG glomerular deposition from the mice that transferred with wt bone marrow-derived dendritic cell (BMDC)-activated lymphocyte-derived DNA (ALD-DNA), the lupus mice were intraperitoneal injected with mouse Ig or 6H3 on the same day after the BMDCs transferred. The mice were sacrificed on week 20 after the BMDCs transferred. Representative result from each group of five mice is shown. Values represent means ± SEM (**p* < 0.05, ***p* < 0.005, ****p* < 0.0005).

Figure S4 **(A)** The half-life of B7-H4 Ig in mice serum after dynamic hydro-injection of B7-H4 IgV plasmid is shown. **(B)** Total IgG against ds-DNA in serum from WT BALB/C mice transferred with normal BALB/C bone marrow-derived dendritic cell (BMDC)-activated lymphocyte-derived DNA (ALD-DNA) or in the serum from **(C)** B7-H4 knockout (B7-H4-KO) mice transferred with B7-H4-KO BMDCs-ALD-DNA. **(D)** Total IgG against ds-DNA in serum of WT or B7-H4-KO mice transferred with WT or B7-H4-KO BMDCs incubated with ALD-DNA. **(E)** Q-PCR analysis of mouse B7-H4 mRNA relative level from BMDCs with specific primers is shown. **(F)** The component of inflammation cell subsets ratio in lupus mice peripheral blood that received B7-H4Ig or control flag treatment. Representative result from each group of 5 mice is shown. Values represent means ± SEM (**p* < 0.05, ***p* < 0.005, ****p* < 0.0005).

Figure S5 **(A)** The total splenocytes of spleens or lymph nodes from B6-lpr/lpr or B7-H4 knockout (B7-H4-KO)-B6-lpr/lpr mice at the age of 30th week. **(B)** The component of pro-inflammation cell subsets ratio in B6-lpr/lpr mice or B7-H4-KO-B6-lpr/lpr mice at the age of 20th week is shown. **(C)** The component of Tfh cells in the spleen and the Tregs cell subsets ratio in the peripheral blood of the B6-lpr/lpr mice or B7-H4-KO-B6-lpr/lpr mice at the age of 20th week is shown. **(D)** Total IgG against ds-DNA in sera of normal C57BL/6 mice treated with mIg or 6H3 as the same dosage of the B6-lpr mice. Representative result from each group of five mice is shown. Values represent means ± SEM (**p* < 0.05, ***p* < 0.005, ****p* < 0.0005).

Figure S6 6H3 or B7-H4 Ig did not influence the normal WT mouse CD4^+^ T cells proliferation, but they affected the bone marrow-derived dendritic cell (BMDC)-activated lymphocyte-derived DNA (ALD-DNA) induced lupus mice CD4^+^ T cells proliferation. Normal WT BMDCs were incubated with ALD-DNA 12 h and then co-cultured with CD4^+^ T cells from normal WT mice spleen or BMDC-ALD-DNA induced lupus mice spleen, 48 h later test the cells proliferation with CCK8 kit. Values represent means ± SEM (***p* < 0.005, ****p* < 0.0005).

Figure S7 Conclusion: there existed a cycle in lupus mice, dendritic cells (DCs) recognized the pathologic DNA from the cell damage through the TLRs on the surface, then the DCs delivered the immuno-signals to CD4^+^ T cells, the CD4^+^ T cells then promoted the B cells to autoantibodies production or directly produced pathology and further caused cell damages and released auto-DNAs on systemic circulation, thus these damages will cause the pathologic DNAs releasing, and be recognized by DCs again. B7-H4 expressed on DCs and incorporation with his unknown receptor on CD4^+^ T cells, and then inhibited the CD4^+^ T cells function to alleviate systemic lupus erythematosus.
