# Supplementary material for: Immunosuppressive Effect of B7-H4 Pathway in a Murine Systemic Lupus Erythematosus Model
Source: Front Immunol. 2017 Dec 11;8:1765. doi: 10.3389/fimmu.2017.01765 (PMC5732181; doi:10.3389/fimmu.2017.01765)

Supplement figure 1

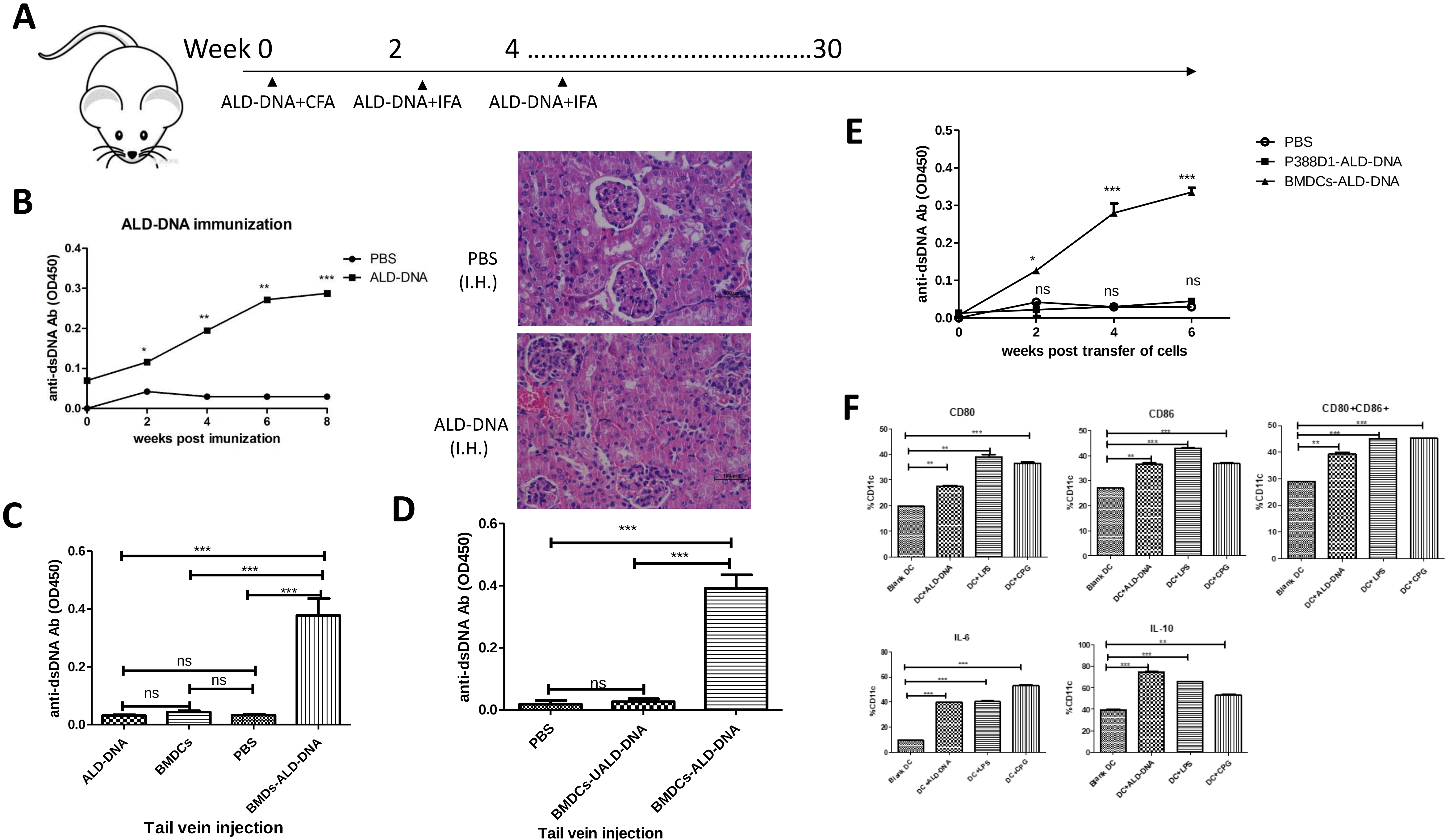

Supplement figure 1

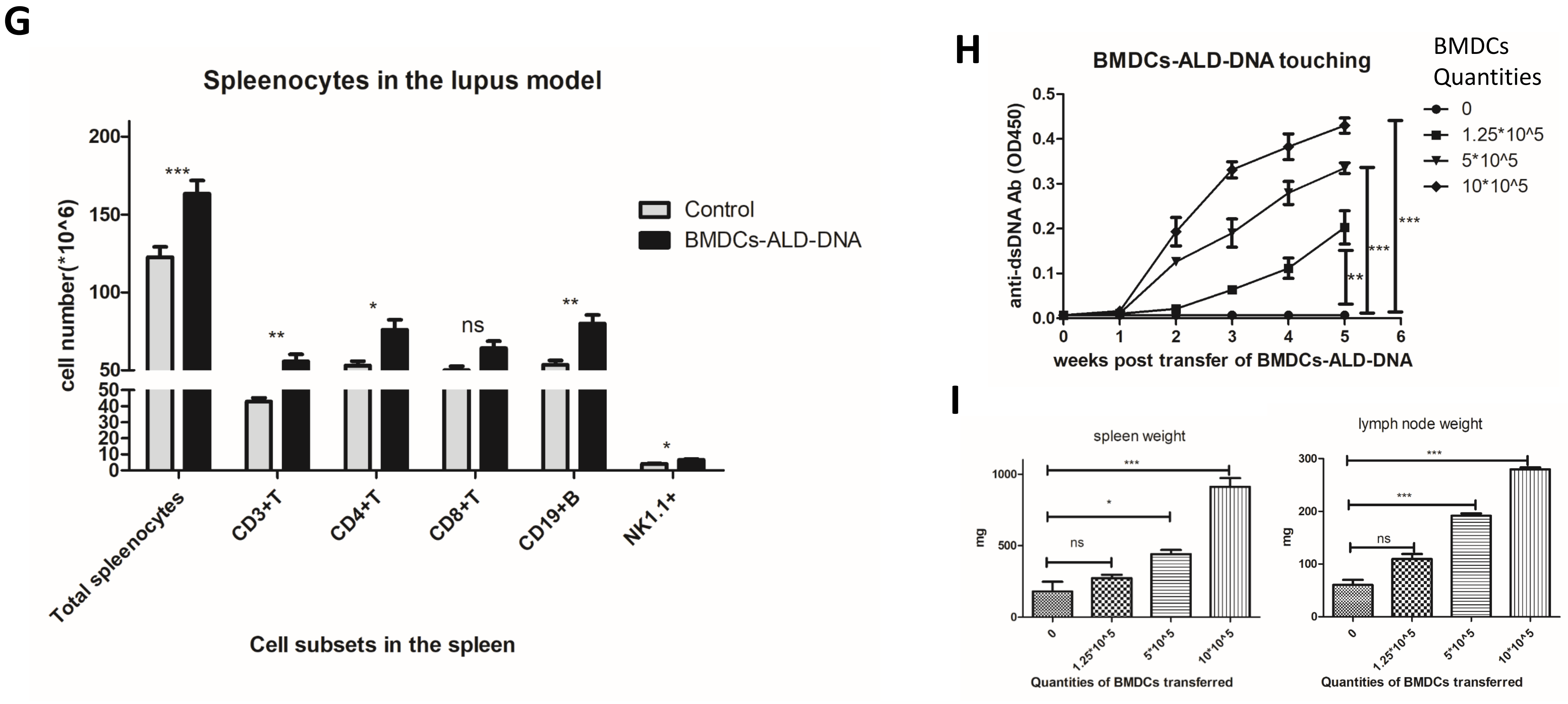

Supplement figure 2

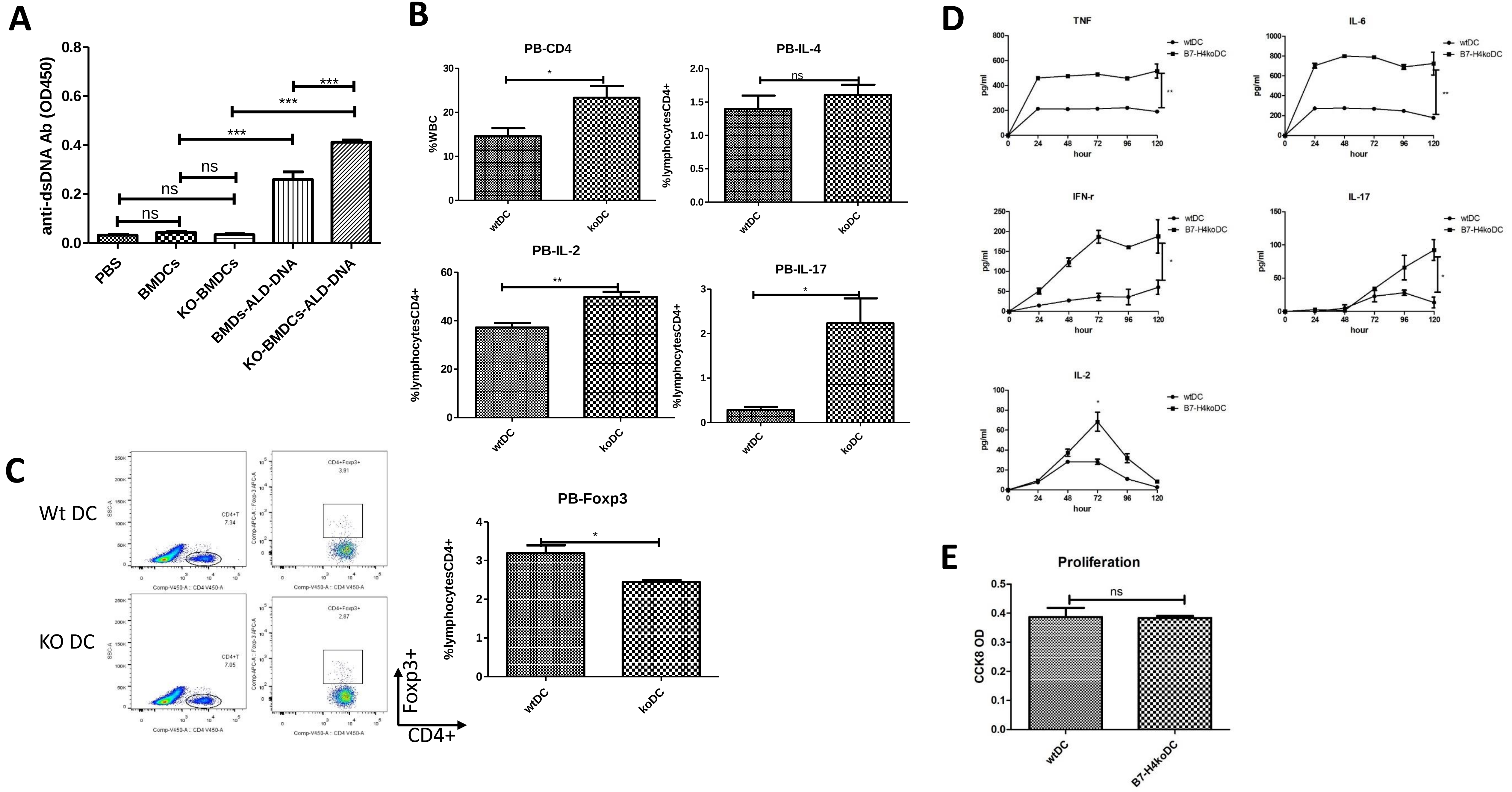

Supplement figure 2

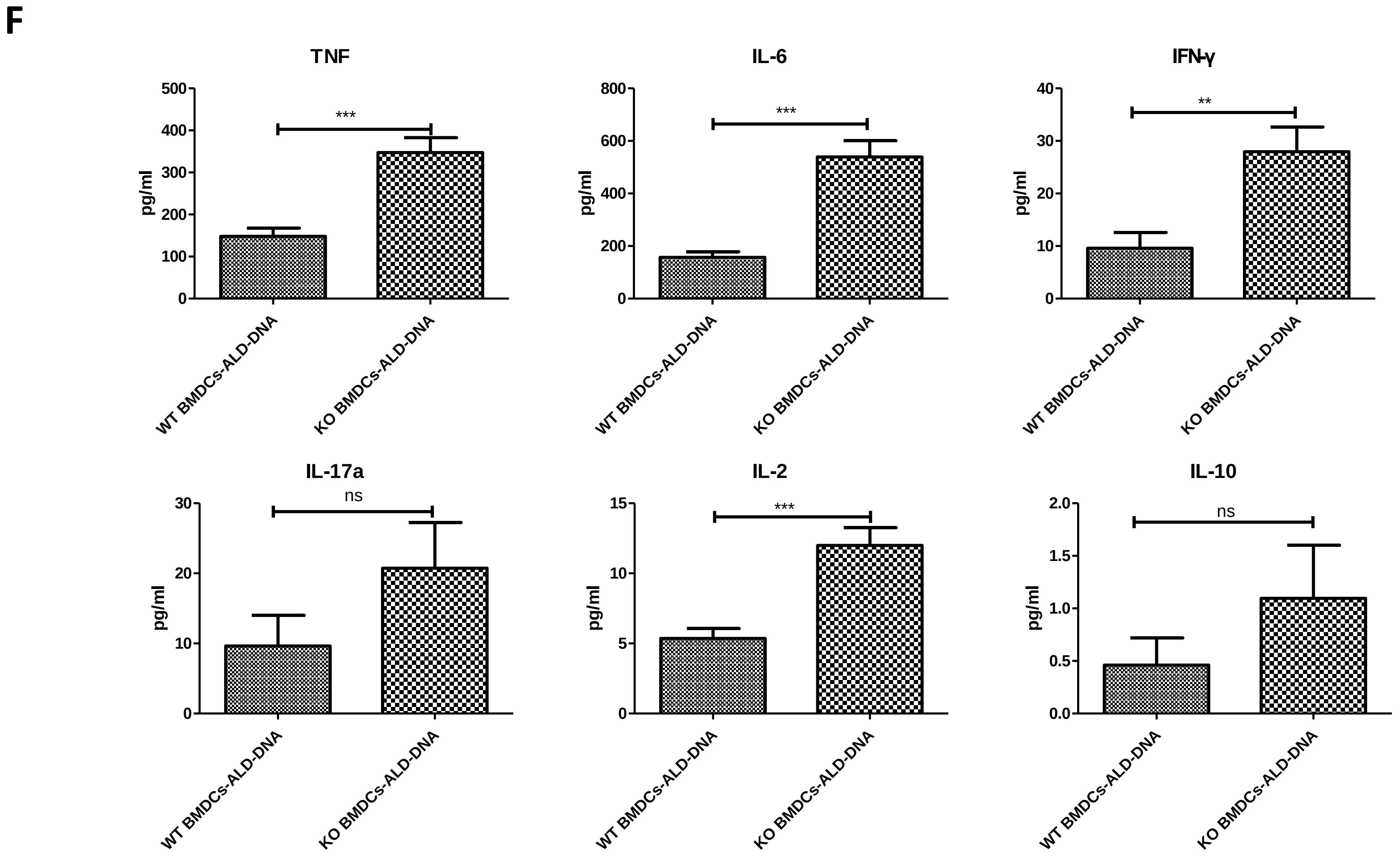

Supplement figure 3

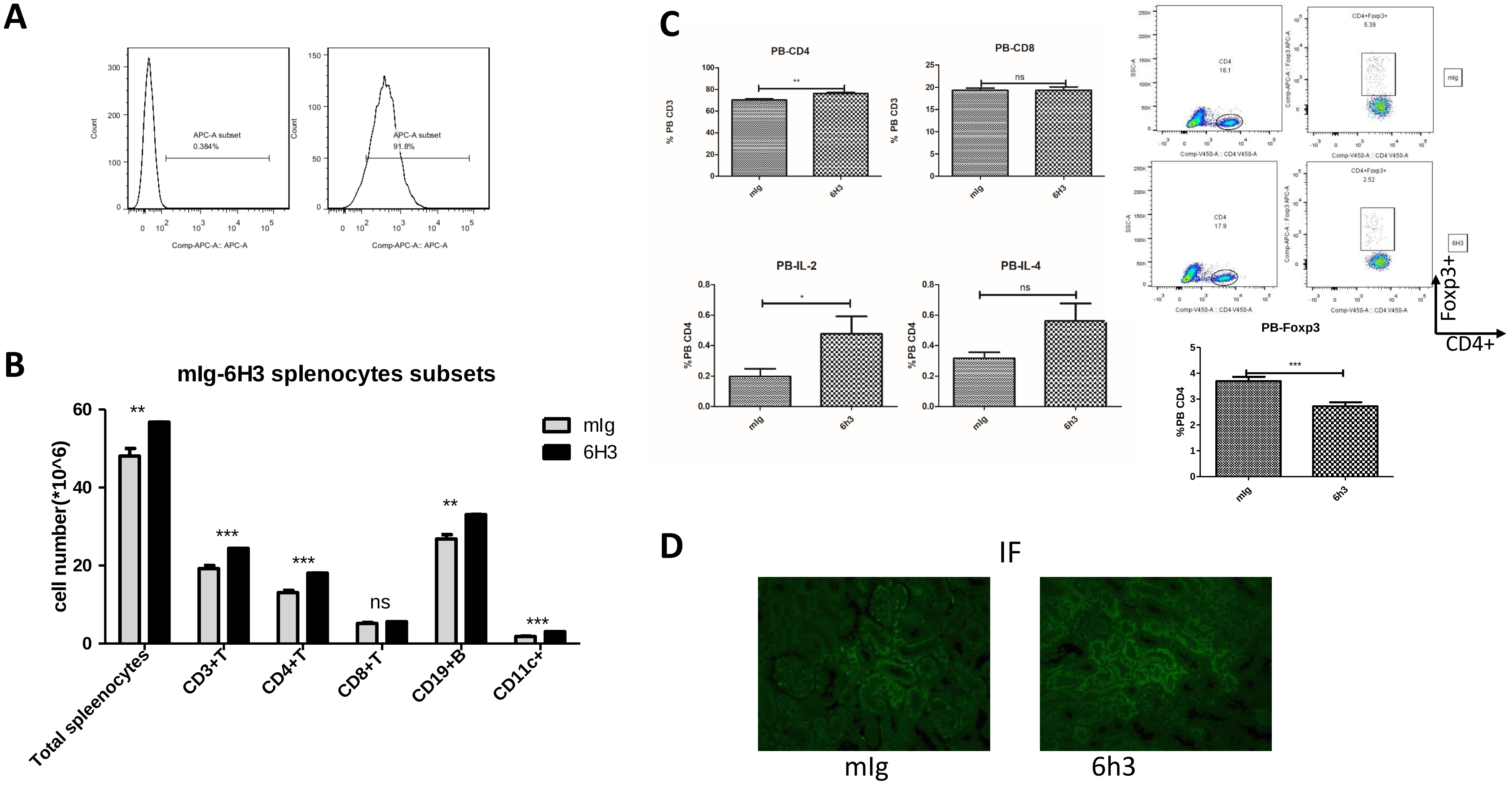

Supplement figure 4

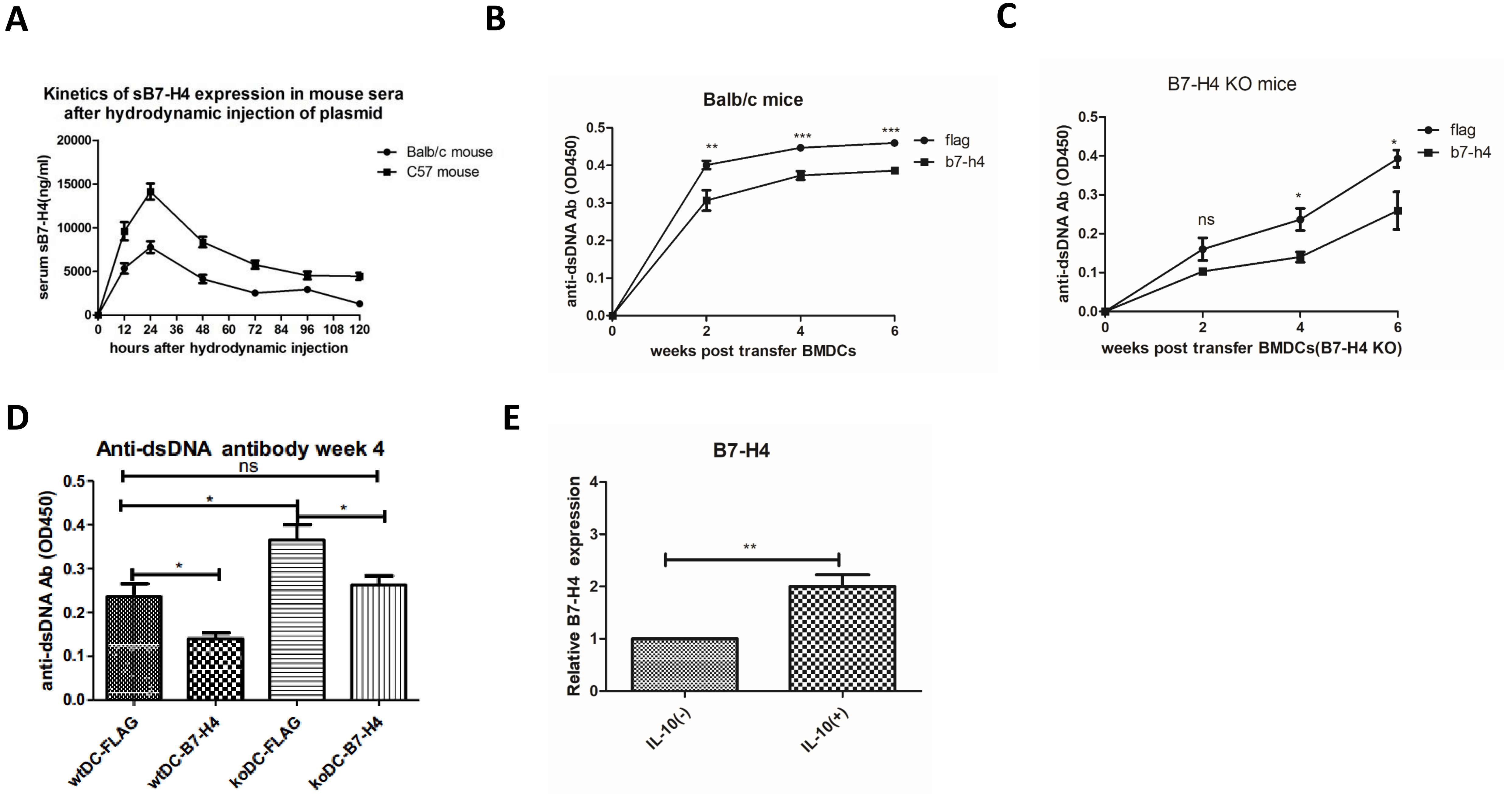

Supplement figure 4

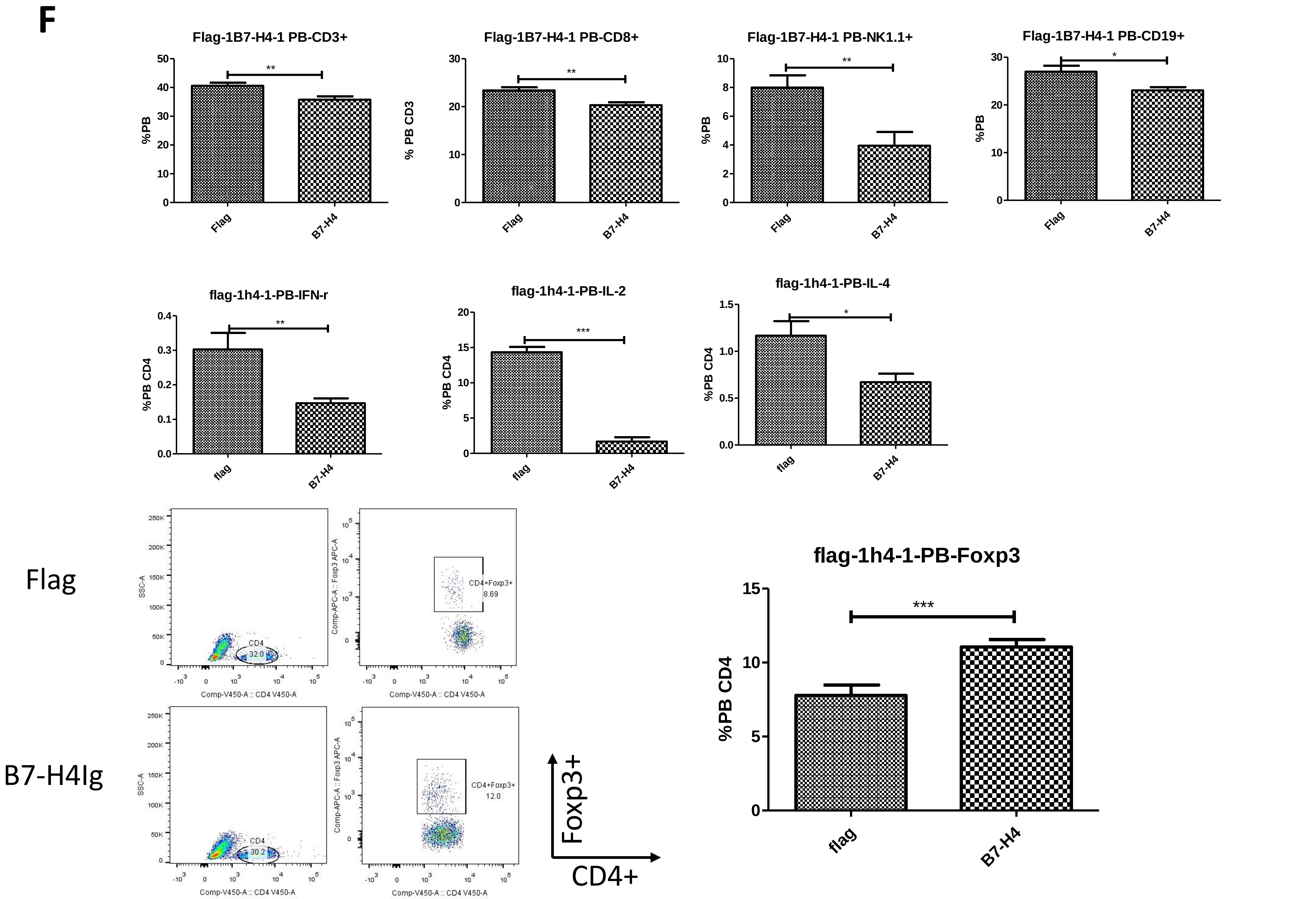

Supplement figure 5

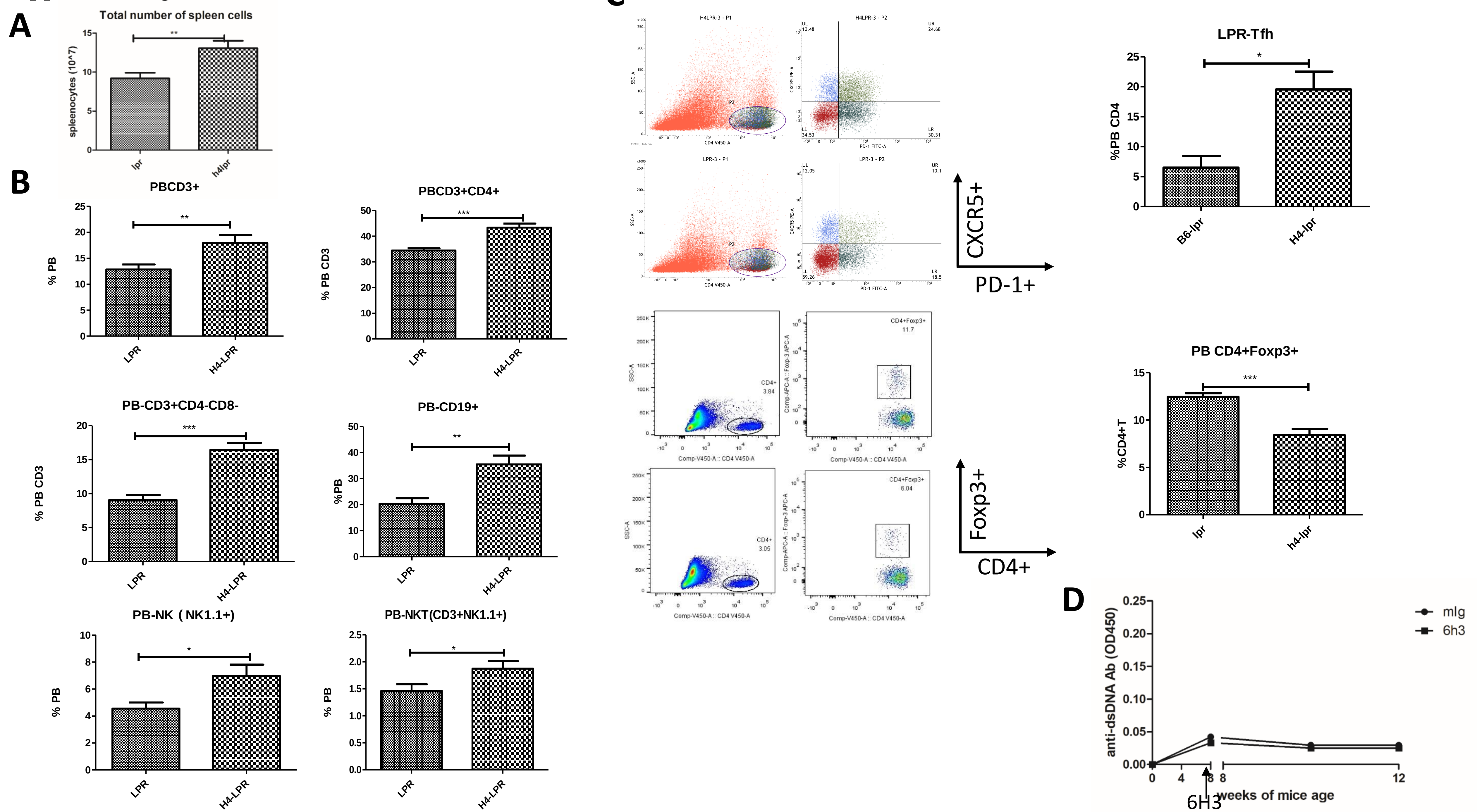

Supplement figure 6

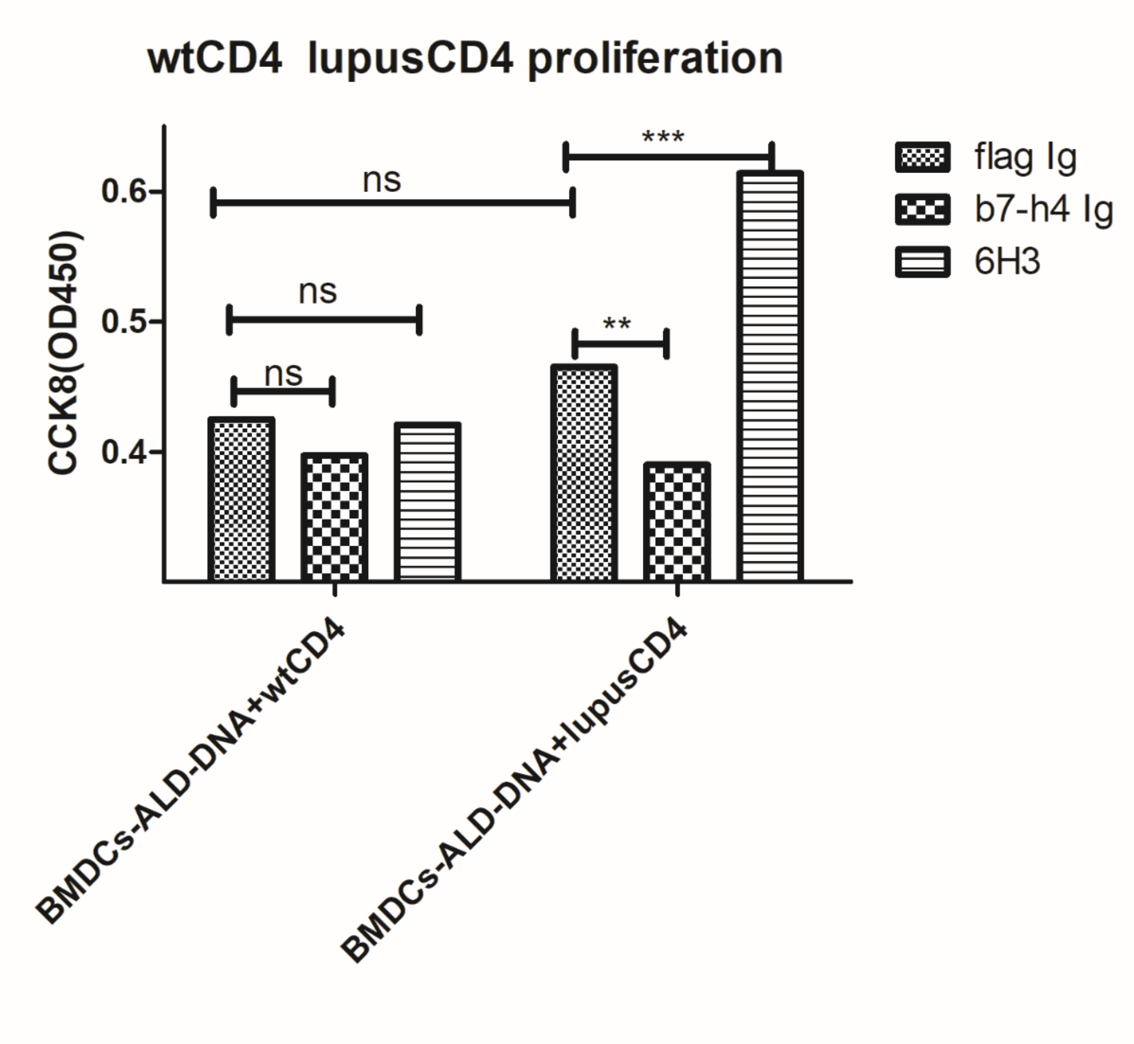

Supplement figure 7

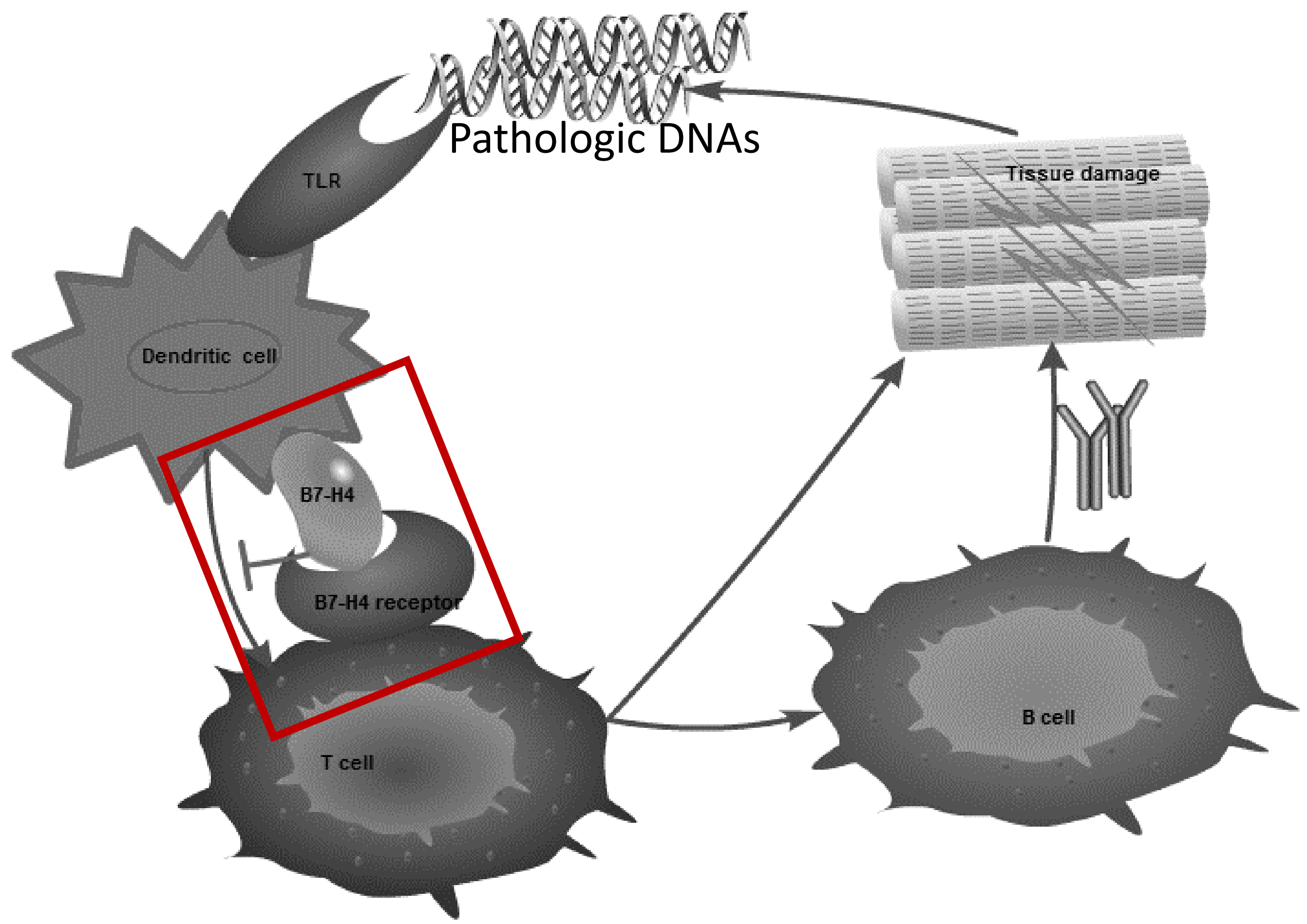

Supplement: Figure S1 — (A) Flow chart for the normal C57BL/6 mice immunized subcutaneously with activated lymphocyte-derived DNA (ALD-DNA). (B) Total IgG against ds-DNA in sera (left) and the histology of the kidney (right) from the mice that immunized subcutaneously with PBS or ALD-DNA. (C) Total IgG against ds-DNA in sera of normal wild-type C57BL/6 mice transferred with PBS, bone marrow-derived dendritic cells (BMDCs), ALD-DNA, or BMDCs-ALD-DNA through tail vein on week 4 post transferred. (D) Total IgG against ds-DNA in sera of normal wild-type C57BL/6 mice transferred with PBS, BMDCs-UALD-DNA, or BMDCs-ALD-DNA on week 4 post transferred. (E) Total IgG against ds-DNA in sera of normal wild-type C57BL/6 mice transferred with PBS, BMDCs-ALD-DNA, or P388D1-ALD-DNA. (F) The changes of the BMDCs after incubated with ALD-DNA, LPS or the widely accepted TLR9 ligand CPG for 24 h using FACS. (G) Total splenocytes and cell subsets numbers in the spleen between control mice and the BMDCs-ALD-DNA transferred mice. (H) Total IgG against ds-DNA in sera and the spleen or lymph node weight (I) of normal WT C57BL/6 mice transferred with BMDCs-ALD-DNA in different quantities (0, 1.25 × 10^5^, 5 × 10^5^, 10 × 105). Representative result from each group of five mice is shown. Values represent means ± SEM (*p < 0.05, **p < 0.005, ***p < 0.0005). [file Data_Sheet_1.PDF]
